# Evaluation of nutrient composition and bone-promoting activity of miiuy croaker (*Miichthys miiuy*) bone

**DOI:** 10.3389/fnut.2024.1510028

**Published:** 2024-12-31

**Authors:** Qianqian Ouyang, Lifen Liu, Lili Liu, Yi Li, Yi Qi, Kefeng Wu, Guoping Zhu, Hua Ye

**Affiliations:** ^1^School of Ocean and Tropical Medicine. Guangdong Medical University, Zhanjiang, Guangdong, China; ^2^Guangdong Provincial Key Laboratory of Aquatic Product Processing and Safety, College of Food Science and Technology, Guangdong Ocean University, Zhanjiang, China; ^3^The Marine Biomedical Research Institute of Guangdong Zhanjiang, Zhanjiang, China; ^4^The Affiliated Hospital of Guangdong Medical University, Zhanjiang, China

**Keywords:** *Miichthys miiuy* bone, nutrient composition, peptide, bone-promoting activity, osteoporosis

## Abstract

**Introduction:**

The objective of this study was to improve the economic value of the processed by-products of farmed miiuy croaker (*Miichthys miiuy*) by evaluating the nutrient composition and osteogenic activity of its bones. We prepared *Miichthys miiuy* bone peptides (MMBP) and analyzed their osteogenic potential.

**Methods:**

We assessed the osteogenic activity of MMBP by molecular docking, MC3T3-E1 cell proliferation assay and zebrafish growth model, and evaluated its effect on osteoporosis (OP) using a retinoic acid-induced osteoporosis rat model.

**Results:**

*Sciaena ossificans* bone is rich in nutrients, including 11.40% water, 59.30% ash, 1.60% crude fat, 27.10% crude protein, and 0.58% total sugars. The total amino acids account for 22.13%, including 4.33% essential amino acids and 17.80% non-essential amino acids. The mineral content was rich, with calcium, phosphorus and selenium contents of 162511, 7151, and 0.264 mg/kg, respectively. MMBP significantly promoted the proliferation of MC3T3-E1 cells, facilitated the growth and bone development of zebrafish. In retinoic acid-induced osteoporosis rat model, increased the serum calcium and phosphorus levels, attenuated the calcium loss, and reduced the tartrate-resistant acid phosphatase and alkaline phosphatase (ALP) activities and significantly improved bone density. MMBP shows potential as a functional food ingredient due to its osteogenic properties, which may help promote bone growth and maintain bone health. These findings provide a scientific basis for the high-value utilization of *Miichthys miiuy* by-products and a new direction for the development of novel functional food ingredients.

## 1 Introduction

Osteoporosis (OP) is a systemic bone metabolism disease of old age, which mainly manifests as a decrease in bone content and quality, the destruction of the fibrous structure of bone tissue, and an increase in bone fragility and fracture (1). OP has become one of the most common diseases in individuals older than 50 years, involving more than 200 million people worldwide, and its incidence is steadily increasing (2). Osteoporotic fractures lead to a high patient mortality rate of 20–40%. Therefore, OP treatment and prevention is a public health problem jeopardizing human health (3).

Bone remodeling is based on the coordinated activity of osteoclasts, which resorb old or damaged bone, and osteoblasts, which produce and store new bone matrix (4). Osteoblast-mediated bone formation and osteoclast-mediated bone resorption are closely linked processes in which both cell types constantly communicate with each other to maintain bone homeostasis (5). A primary cause of OP is the dynamic imbalance between osteoblasts and osteoclasts. Drugs that inhibit bone resorption and or increase bone formation have been developed for the treatment of OP, including bisphosphonates, estrogens, calcitonin, and fluoride supplements. However, chronic usage of these medications may result in adverse effects like sciatica, elevated breast cancer risk, and gastrointestinal issues (6). Therefore, searching for functional biocompounds that are natural and nontoxic is an effective strategy for the prevention and treatment of OP (7).

Current therapeutic approaches rely on dietary interventions as a cost-effective strategy to improve bone health and reduce fracture risk (8). Proteins or peptides can either promote osteoblast proliferation and differentiation or inhibit osteoclast differentiation to increase bone formation and decrease bone resorption. For instance, lactoferrin peptides promote osteoblast growth and reduce osteoclast differentiation *in vitro* (9). Given the reported association between consumable mammalian collagen peptides and the increased risk of zoonotic diseases, there has been a traditional reliance on livestock animal tissues, such as those from cattle and pigs, for commercial collagen peptide derivation. However, using collagen peptides from terrestrial animals carries a risk of disease transmission, including spongiform encephalopathy and foot-and-mouth disease, and may trigger immune reactions in humans, affecting approximately 3% of the population (10). Moreover, consuming collagen from conventional sources is restricted by religious convictions and practices (11). Marine collagen has garnered interest owing to its general safety and minimal immunogenicity. Many potentially active peptides have been isolated from marine organisms, usually consisting of 2–20 amino acids, and have attracted much attention from the academic and healthcare communities due to their excellent absorption properties and bioactivities, such as antioxidant, anti-inflammatory, and anti-hypertensive activities (12). More importantly, active peptides of marine origin have shown good osteogenic effects both *in vitro* and *in vivo*, and their anti-osteoporotic properties have been gradually explored in recent years. Some collagen peptides derived from marine sources, such as Antarctic krill (13) and blue mussel peptides (14), have been shown to improve OP, enhance calcium (Ca) utilization, and increase bone density.

Because of its high nutritional content, appealing flavor, and significant economic significance, *Miichthys miiuy*, commonly known as the brown croaker, which is primarily found in the western Sea of Japan to the East China Sea, has been extensively cultivated in China since the late 1990s. Fish bones are frequently discarded during *M. miiuy* processing, producing enormous amounts of waste (15). Byproduct accumulation has become a serious issue and causes environmental challenges (e.g., water pollution and greenhouse gas emission) worldwide. However, fish bones are rich in calcium, phosphonium (P), and other trace elements required by the human body, as well as proteins, amino acids, and other nutrients, suggesting the potential for high-value utilization of aquatic product processing byproducts in recent years. Collagen extracted from fish bone has low immunogenicity and is water soluble, biocompatible, and biodegradable (16). For instance, the peptide DGPSGPK purified from tilapia bone hydrolysate have a considerable effect on MC3T3-E1 cell proliferation and differentiation (17) and promote bone formation. Peptides from Pacific cod bone improve calcium utilization and increase calcium in rat femurs (18). Therefore, developing peptides containing active amino acids from fish bone may be important for osteogenic differentiation.

Although a sizable amount of fishbone byproducts are produced annually, fishbone can be used effectively to maximize its biological value and generate additional economic advantages. Given that the production of peptides with active amino acids is essential for osteogenic development, many studies have attempted to extract proteins and peptides from fish bones to evaluate their osteogenic activity (19–21). In the present study, the nutrient and mineral contents of *M. miiuy* bone were analyzed and the osteogenic activity of peptides from *M. miiuy* bone was assessed using molecular docking, a larval zebrafish model, and validation of the activity of MC3T3-E1 cells based on proliferative culture *in vitro*. Additionally, we systematically evaluated the pharmacological effects of *M. miiuy* bone peptide (MMBP) on OP using a retinoic acid–induced rat OP model. This study offers new insights into the high-value utilization of osteogenic byproducts and the development of functional diets.

## 2 Materials and methods

### 2.1 Materials and reagents

*M. miiuy* bone was purchased from Yifanghai Ocean Ranch Co (Huizhou, China). The *M. miiuy* bone were frozen and stored at −80°C until further analysis, and they were thawed at room temperature for 1 h before the determination of nutrients. Penicillin and streptomycin double antibody was purchased from Solarbio (Beijing, China). CCK8 was purchased from Beyotime (Shanghai, China). Fetal bovine serum and MC3T3-E1 were obtained from Procell (Wuhan, China). α-MEM medium was purchased from Gibco (Grand Island, USA). Alkaline phosphatase (ALP), and Calcium colorimetric test kits were purchased from Beyotime (Shanghai, China). The Serum phosphorus test kit, anti-tartaric acid phosphatase assay test kit, and Calcium xanthophyll were purchased from Solarbio (Beijing, China).

### 2.2 Nutrient composition analysis of *M. miiuy* bone

#### 2.2.1 Determination of basic nutrients

Moisture content was measured by drying in an oven at 105°C until a constant weight was reached. The Kjeldahl technique was used to determine the protein content. Total nitrogen was multiplied by 6.25 to determine the crude protein content (22). The Soxhlet extraction method was used to determine the fat content (23). The phenol sulfate technique was used to calculate the total sugar content, and cauterization and weighing were employed to quantify the ash content (24).

#### 2.2.2 Determination of amino acid content and nutritional evaluation

The technique of Domínguez et al. (25) was used to determine the amino acid content. *M. miiuy* bone was crushed into powder, 100 mg of powder was weighed, hydrolysed with 5 ml of 6 M HCl acid and heated at 110°C for 24 h. Amino acids were quantified using an amino acid analyzer (Biochrom, UK).

Amino acid score (AAS) and chemical score (CS) were used for amino acid nutritional evaluation (26).

Calculations:


(1) $$\text{m}=\frac{\text{n}}{{\text{n}}_{\text{pro}}}\times 6.25\times 100$$

(2) $$\text{AAS}=\frac{{\text{m}}_{\text{EAA}}}{{\text{M}}_{\left(\frac{\text{FAO}}{\text{WHO}}\right)}}$$

(3) $$\text{CS}=\frac{{\text{M}}_{\text{EAA}}}{{\text{M}}_{\text{Egg}}}$$

Where:

m: Amino acid content (mg/N);

n: Content of an amino acid in a sample;

n_*Pro*_: Protein content (mg/N);

M_*EAA*_: The amount of a certain essential amino acid (EAA) in the measured protein sample (mg/N);

M (FAO/WHO): Content of certain EAAs in the FAO/WHO scoring benchmarks (mg/N);

M_*Egg*_: Content of certain EAAs in egg protein (mg/gN).

#### 2.2.3 Mineral element analysis

The samples were microwave-digested, filtered, and analyzed for calcium, selenium, and phosphonium via inductively coupled plasma emission spectrometry (Thermo Fisher, USA) (27). Plasma power: 1150 W, carrier gas flow rate: 5,000 mL min^–1^, cooling gas flow rate: 12 000 mL min^–1^, auxiliary gas flow rate: 500 mL min^–1^, peristaltic pump speed: 50 r min^–1^.

### 2.3 Preparation, characterization, and computerized virtual screening of MMBP

#### 2.3.1 Preparation and characterization of MMBP

MMBP was prepared according to the method by Tan et al. (22). *M. miiuy* bone was mixed with Milli-Q wate in a ratio of 1:3 (w/v) and extracted for 90 min at 121°C ± 1°C in an oil bath. To gather the supernatant, the resultant mixture was filtered and centrifuged at 8,000 × *g* for 15 min. Freeze-dried collagen by Freeze dryer (BUCHI Switzerland) was stored at −20°C. *M. miiuy* bone collagen was mixed with ultrapure water at a ratio of 3:100 (w/v). Flavored protease at a ratio (E:S) of 1:50 (w/w) was added to the *M. miiuy* bone collagen and hydrolyzed at pH 6.5 and 55°C for 3 h. Inactivation was carried out in boiling water for 10 min. Subsequently, the mixture was centrifuged at 8,000 × *g* for 15 min, and the supernatant was collected, freeze-dried, and stored at −20°C. The sample was desalted using a Sep-Pak C18 desalting column and dried using a centrifugal concentration dryer. Next, NanoDrop 2000 was used to determine the concentration of the peptides dissolved in distilled water. The samples were first dissolved with 50 mM NH_4_HCO_3_, then reduced, alkylated, and desalted, after which the processed samples were analyzed with liquid chromatography–tandem mass spectrometry (LC–MS/MS) (LC: Easy-nLC 1200 Thermo Fisher Scientific; Q Exactive Hybrid Quadrupole-Orbitrap Mass Spectrometer, Thermo Fisher Scientific). Capillary liquid chromatography conditions were mobile phase A: 0.1 per cent formic acid, mobile phase B: 0.1% formic acid, 80% CAN, flow rate: 600 nL/min. Primary mass spectrometry parameters were: Resolution: 60,000, AGCtarget: 4e5; MaximumIT: 50 ms, Scanrange: 350 to 1,500 m/z. Secondary Mass Spectrometry Conditions were resolution: 15,000, AGCtarget: 5e4, MaximumIT: 22 ms; TopN: 20; NCE/steppedNCE: 28.

Analysis time for each component: 120 min the mass spectrometry raw results were queried in the PEAKS Studio database to identify the peptides.

#### 2.3.2 Bioinformatics analysis of MMBP peptides

The online program PeptideRanker^1^ was utilized to predict the bioactivity of the MMBP peptides, where a value of > 0.5 indicated bioactivity. CPPpred^2^ was used to predict the cell permeability of the peptides. PeptideRanker score > 0.5 and CPPpred score > 0.2 were used as screening criteria.

Toxin Pred^3^ was used to predict the toxicity of the peptides. The allergenicity of the peptides was predicted with the Aller TOP v.2.0 program.^4^ The Innovagen website^5^ was used to predict water solubility. Peptides predicted to be nontoxic-nonsensitizing, and water soluble were used for subsequent studies.

The amino acid sequences of the screened peptides were submitted to the BIOPEP database^6^ to search for matching known functional peptides.

The relative molecular mass and isoelectric point of the peptides were calculated using the Expasy-compute PIMw tool^7^. Pepdraw^8^ was used to forecast the net charge of the peptides, and ProParam^9^ was used to predict their instability index.

#### 2.3.3 Molecular docking

The bone morphogenic protein (BMP)-2/SMAD signaling pathway is widely recognized for its crucial role in osteogenesis. BMPs activate the SMAD signaling pathway by adhering to the surface of cells expressing BMP receptors. This process triggers the translation and transcription of responsive genes. These sensitive genes include transcription factors associated with osteoblast development, such as Runx2, osterix, collagen I, and other bone matrix proteins (28). Furthermore, BMPs impact osteoblast differentiation via non-SMAD signaling pathways, including MAPK, PI3K/Akt, and Wnt/β-catenin (29).

BMPR1A, a key receptor in the BMP-2/SMAD signaling pathway axis (30), is particularly instrumental in these events. Upon BMP-2 binding and activation of BMPR1A, SMAD proteins are phosphorylated and activated, leading to the upregulation of osteoblast-specific genes and biomineral deposit formation. This pathway is targeted in molecular docking studies aimed at identifying macromolecular bone phosphopeptides with potential therapeutic effects on OP.

To facilitate these studies, ChemBio3D Ultra 14.0 was utilized to construct the peptide sequences. The three-dimensional structure of BMPR1A was accessed through the Protein Data Bank with the identifier 2h62.^10^ Employing AutoDockVina, the ligand was docked with the protein. The crystal structure of the target protein was created by eliminating water molecules and adding hydrogen atoms before docking. The grid map’s center was positioned over the protein’s active site, with the grid box dimensions set to 80 Å × 80 Å × 80 Å in order to encompass the interaction domain. The resulting affinity (kcal/mol) value indicates the binding affinity between the ligand and receptor. A lower affinity value signifies stronger binding interactions, suggesting a more effective therapeutic compound.

### 2.4 Effect of MMBP on MC3T3-E1 proliferation

In an incubator set at 37°C with 5% CO_2_, mouse preosteoblast cell line MC3T3-E1 cells were grown in α-MEM supplemented with 1% penicillin double antibiotic solution and 10% fetal bovine serum. The cells were grown in a cell culture flask after digestion with 0.25% trypsin–EDTA when they reached 80–90% confluence. The CCK8 assay was used to measure cell proliferation. The cells were then injected into 96-well plates at a density of 5 × 10^3^ cells/well and incubated for 24 h. The blank zeroing group only contained added culture medium, the control group consisted of cells without MMBP, and the experimental groups contained different concentrations of MMBP (0, 10, 50, 100, 200, 500, 800, and 1,000 μg/mL). The cells were incubated for 24 and 48 h. Subsequently, 10 mL of CCK8 was introduced to each well, followed by incubation for 2 h. The absorbance of each well was calculated at 450 nm using an enzyme meter.

Cell proliferation was calculated according to the following formula:


(4) Cell⁢proliferation=experimental⁢groupA450-blank⁢groupA450control⁢groupsA450-blank⁢groupA450

### 2.5 Effect of MMBP on the length and skeletal development of zebrafish larvae

#### 2.5.1 Zebrafish rearing

Zebrafish AB lines, purchased from Hangzhou Huante Biological, Laboratory Animal Use License No. SYXK (Zhejiang) 2022-0004, were accredited by the Association for Assessment and Accreditation of Laboratory Animals International (AAALAC). They were housed in a 28.5°C circulating water tank with a 14:10-h day:night cycle. Fertilized from natural spawning, they were gathered and incubated at 28.5°C in embryonic water with 2 mg/mL methylene blue.

#### 2.5.2 Zebrafish body length determination

Zebrafish larvae were randomly transferred into 12-well plates, with no fewer than 10 fish in each group. At 3 days postfertilization (dpf), they were treated with 0, 10, 50, or 100 μg/mL MMBP to determine the growth rate. Fresh MMBP was added to the media each day. The zebrafish were cultured to 6, 9, and 12 dpf, anesthetized with 0.02% tricaine, and their lengths were measured using an Olympus stereomicroscope.

#### 2.5.3 Evaluation of zebrafish juveniles for osteogenesis

In zebrafish larvae, Ca xanthophyll green fluorescent labeling was utilized to track bone production. Zebrafish larvae were transferred into 12-well plates with no fewer than 10 fish per well, and 0, 10, 50, and 100 μg/mL of MMBP was added at 3 dpf. Fresh MMBP was added to the media each day. The larvae were rinsed three times with embryonic water for 10 min each, after which they were submerged in a 0.2% Ca xanthophyll solution for 10 min at 9 dpf. A laser confocal microscope was used to observe the fluorescence signal of the zebrafish vertebrae. The fluorescence intensity of vertebral bone volume was quantified using Image J morphological analysis software.

### 2.6 Preventive effects of MMBP in a rat model of retinoic acid–induced OP

#### 2.6.1 Animal groups and drug administration

Three-month-old healthy SD female rats (200–220 g) were commercially supplied by SiPeiFu Biotechnology Co (Beijing, China), License No SCXK2019-0010. All animal procedures were performed in accordance with the guidelines and ethics approval of Experimental Animal Care and Use Committee of Guangdong Medical University. The animals were randomly assigned to five groups, each consisting of eight rats: MMBP high-dose (H) group, MMBP low-dose (L) group, model control (MC) group, positive control (PC) group, and normal control (NC) group. After one week of acclimatization feeding, except for the NC group, the rats of other groups were administered 75 mg/kg bw/day (d) of retinoic acid by gavage to establish the OP model after 2 weeks of ongoing gavage. The rats in each group were housed in separate cages, fed standard diets, and given free access to water during the treatment period. After 2 weeks, retinoic acid gavage was stopped. Four weeks of 0.9% saline was administered to the NC and MC groups, alendronate 5 mg/kg bw/d was given to the PC group, and the L and H groups were gavaged with 100 and 300 mg/kg bw/d, respectively. Bedding was changed daily, and body weight recorded weekly (31). Following the final dosage, the rats were fasted for 12 h and then anesthetized with Avertin, after which blood samples were collected from the heart. The serum was separated by centrifugation and kept at −80°C. Then, the rats were euthanized by cervical dislocation. The heart, liver, spleen, lungs, kidneys, ovaries, and other organs were removed and weighed. The femur and tibia from both sides were excised and the muscles and soft tissues were taken out. These femur and tibia were rinsed with 0.9% saline and stored at −80°C for subsequent studies.

#### 2.6.2 Serum calcium, phosphonium, ALP, and tartrate-resistant acid phosphate (TRAP) measurements

Rats’ Serum calcium, levels were measured using the colorimetric o-cresolphthalein complexone (OCPC) technique. Rats’ serum calcium, levels were measured using the colorimetric o-cresolphthalein complexone (OCPC) technique. Calcium, react with OCPC under alkaline conditions to produce a purple complex, and absorbance at 575 nm can be used to measure the calcium, concentration.

Serum phosphonium levels of rats were determined using the phosphomolybdic acid method. Molybdic acid and inorganic phosphonium in the serum samples combine to generate phosphomolybdic acid, which is reduced to molybdenum blue, with a maximum absorption peak occurring at 660 nm.

Disodium benzene phosphate was broken down by serum ALP to yield free phenol and phosphoric acid. Under alkaline circumstances, the free phenol interacted with 4-aminoantipyrine, which was subsequently oxidized by potassium ferricyanide to produce a crimson quinone derivative. Within a certain concentration range, the color shade was positively correlated with ALP activity in the rat serum samples. The optical density (OD) values were determined at 520 nm using an enzyme marker, and sample concentrations were determined by comparing with a standard concentration of phenol stock solution.

TRAP activity was determined according to the kit instructions. Para-nitrophenyl phosphate is a commonly used phosphatase chromogenic substrate that produces para-nitrophenol under acidic conditions in the presence of acid phosphatase. Para-nitrophenol appears as a yellow product under alkaline conditions and can be detected by absorbance under alkaline conditions. By measuring the absorbance at 405 nm, para-nitrophenol can be identified The yellow product as TRAP activity increases, and TRAP activity can be calculated by colorimetric analysis.

#### 2.6.3 Bone mineral density (BMD) measurement

The left tibia of each rat was placed on the measurement platform of an X-ray small animal bone densitometer (U1traFocus DXA, USA) and scanned directly for left tibial bone density.

### 2.7 Data analysis

Data are expressed as mean ± standard error of mean (SEM). One-way analysis of variance was used to analyze the experimental data. *p* < 0.05 was recognized as a statistically significant difference, and the experimental data were statistically analyzed using GraphPad 8.3.0 software.

## 3 Results and discussion

### 3.1 Basic nutrient composition of cultured *M. miiuy* bone

*M. miiuy* bone is a by-product produced during the processing of Sciaena food products. As shown in Table 1, *M. miiuy* bone had significantly higher protein and ash content than the bones of sea bass, rainbow trout, and gilthead sea bream, whereas the fat content was significantly lower than that of the three types of fish bones. These data show that *M. miiuy* fish bone is a valuable source of protein and polysaccharides. These biocompounds demonstrate remarkable antioxidant anticoagulant, anticlotting, hypoglycemic, and weight loss activities (32–35). Sulfated heteropolysaccharides extracted from fish bones, skin, and heads of tuna byproducts exhibited varying degrees of antimicrobial and antioxidant activities, which those isolated from fish bones showing the highest activity. The differences were mainly due to the diversity of naturally occurring sugar chains, which could have differed in sulfate content and molecular weight redistribution, and the fact that fish bones had the highest levels of total sugars, glyoxylates, and sulfate groups may indicate that they are highly bioactive (36).

**TABLE 1:** Nutrient content of different fish bones (g/100 g).

Fish bone type	Padding	Protein	Total sugar	Ash	Fat
*Miichthys miiuy*	11.40	27.10	0.58	59.30	1.6
Sea bass (23)	52	15	–	7	19
Rainbow trout (37)	59.6	15.9	–	–	17.7
Gilthead sea bream (38)	55.24	16.39	–	6.23	17.13

“–” indicates that no information is available at this time.

### 3.2 Mineral element analysis

The Ca content (162,511 mg/kg) of *M. miiuy* bone was closer to that of gilthead sea bream, and the P content of 7,151 mg/kg was significantly higher than that of sea bass and gilthead sea bream (*p* < 0.05), as shown in Table 2. Based on the mineral source and the criteria for high content classification, where a high Ca source contains more than 1,600 mg/100 g, and a high P source contains at least 800 mg/100 g, *M. miiuy* bone is a good Ca source (39). Whereas its P concentration was marginally lower than that of P-rich sources, it nonetheless met the criterion for a high P source. Inadequate Ca intake decreases bone mass, leading to OP. Thus, adequate Ca intake is important for maintaining good health and preventing OP (40). Fish bone is a natural source of Ca, and it can provide Ca for organic peptides, which is easy to absorb. Selenium is an essential trace element with important physiological functions such as acting as an antioxidant (41), having anti-inflammatory properties (42), antitumor regulation of thyroid hormone metabolism (43), and regulation of body metabolism (44). The selenium content of *M. miiuy* bone was 0.264 mg/kg, which meets the standard range for selenium enrichment (DB36/T 566-2017). Thus, *M. miiuy* bone can be used as a food source and nutritional supplement rich in Ca and selenium.

**TABLE 2:** Composition of mineral elements and their contents in different fish bones (mg/kg).

Elemental	*Miichthys miiuy*	Sea bass (23)	Gilthead sea bream (38)
Calcium	162,511	2,093	16,188.3
Selenium	0.264	–	–
Phosphonium	7,151	1,166	989.20

“–” indicates that no information is available at this time.

### 3.3 Amino acid analysis

As shown in Supplementary Table 1, sixteen amino acids were detected in *M. miiuy* bone, with a total content of 22.13 g/100 g. The EAA and non-essential amino acid (NEAA) contents were 4.33 g/100 g and 17.8 g/100 g, respectively. *M. miiuy* aspartic acid and glutamic acid are fresh flavor amino acids, and glycine and alanine help enhance the fresh flavor of seafood and impart sweetness (45). The fresh flavor amino acid content was 11.53%, which accounted for 53.45% of the total amino acids. *M. miiuy* bone itself did not possess sweetness and freshness, but it could produce flavor-presenting peptides and free amino acids through digestion and hydrolysis. Different functional groups of amino acid residues interact with taste receptors to produce different flavors, including fresh, sweet, bitter, and astringent (46). Furthermore, the combination of molecular sensory techniques, such as the electronic nose and tongue, can be used to develop *M. miiuy* products with good flavor, thus enriching the utilization value of *M. miiuy* (47). Mechanistic studies have indicated that several amino acids, including five NEAAs (alanine, arginine, glutamic acid, glycine, and proline) contribute to bone health. This contribution is primarily attributed to the enhanced synthesis of collagen and muscle protein as well as increased production of insulin and insulin-like growth factor 1 (48). Thus, *M. miiuy* bone could be used as a high-quality raw material for flavorings and to promote bone health.

As shown in Supplementary Table 2, The nutritional value of the proteins was evaluated with an amino acid score (AAS) and a chemical score (CS). Values closer to 100 represent higher protein nutritional values, determined by how closely the EAA composition matches the scoring model. The first limiting amino acid was isoleucine, with an AAS value of 0.25 and a CS value of 0.19. The second limiting amino acid was leucine, with an AAS value of 0.34 and a CS value of 0.28.

### 3.4 MMBP preparation, characterization, and bioinformatic peptide analysis

#### 3.4.1 Fish bone peptide LC–MS/MS analysis and identification

In total, 366 polypeptides were identified in *M. miiuy* bone via LC–MS/MS. The total ion flow chromatogram is shown in Supplementary Figure 1. Numerous peaks indicated that *M. miiuy* bone collagen comprised a mixture of peptides with different molecular weights. MMBP is mainly composed of glycine, proline, leucine, alanine, glutamate, serine, valine, and threonine. The identified peptide sequences conformed to the typical “G-P-X” sequence of type I collagen α 2, which is very similar to the osteogenic peptide from tilapia bone identified by Liao et al. (21).

#### 3.4.2 Screening of active peptides based on an online database

As shown in Supplementary Table 3, the 366 identified peptides were graded and screened. Twenty-six peptides with a Ranker score of > 0.5, a CPPpred score of > 0.2, nontoxicity, good water solubility, and non-allergenicity were selected. The amino acid sequences of these peptides were entered into the BIOPEP database for comparison, and they were found to be new bioactive peptides (49).

#### 3.4.3 Molecular docking

**FIGURE 1:**
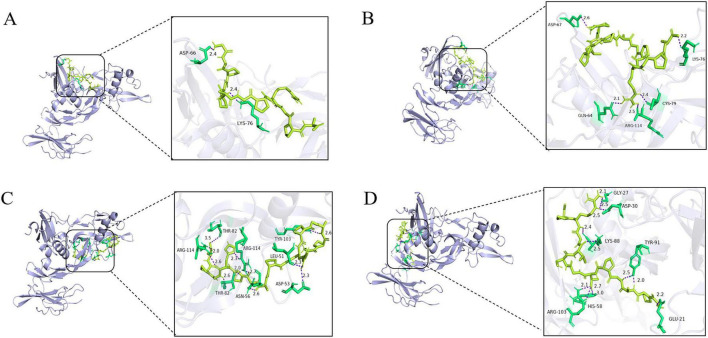
Peptide binding and interacting residues with BMPR1A (ID: 2h62). (P1)- BMPR1A **(A)**, (P2)- BMPR1A **(B)**, (P3)- BMPR1A **(C)**, (P4)- BMPR1A **(D)**, Purple represented hydrogen bonds, yellow represented peptide ligands, and green represents amino acid residues associated with the action.

For the molecular docking of the 26 peptides, we predicted the receptor-ligand docking and binding energies. The more stable the receptor–ligand complex, the lower the docking energy (50). The docking results are shown in Supplementary Table 4. The binding energies of the docked peptides were all < 0, which means that all 26 can bind to the receptor spontaneously (51). Twenty peptides had binding energies less than −7.0, indicating that these peptides were potential compounds for promoting bone formation. According to the score scale, GPAGPRGPAGPHGPA (P3) and GPAGPTGSVGRP (P4) showed strong binding to BMPR1A, both scoring −8.3. GAPGPAGPRGPA (P1), GPAGAPGPAGPRGPA (P2), exhibited high binding to BMPR1A, scoring −8.9, −8.6. As seen in Figure 1, 2 hydrogen bonds form between P1 and the residues ASP-66 and LYS-76 of BMPR1A; P2 forms 5 hydrogen bonds with residues ASP-67, LYS-76, GLN-64, ARG-114, and CYS-79 of BMPR1A; and P3 forms 5 hydrogen bonds with residues THR-82, ARG-114, and ASN-56 of BMPR1A ASP-53, LEU-51TRY-103 residues, 11 hydrogen bonds were observed between P4 and residues of GLY-27, ASP-30, LYS-88, ARG-103, HIS-58, TRY-91, GLU-21d of BMPR1A. The instability indices and electrostatic charges of these peptides were predicted. Peptide permeability across the intestinal epithelium was influenced by several including hydrophobic interactions, molecular size, and charge (52). Peptide stability is important for absorption and their biological activity. When the instability index is < 40, the peptide is considered stable. Two peptides, GPAGAPGPAGPRGPA and GSPGPAGPRGPQGL, had instability indices > 40, indicating that these peptides may be unstable (53). Whether these peptides have osteogenic activity needs to be verified in *in vitro* and *in vivo* experiments.

**FIGURE 2:**
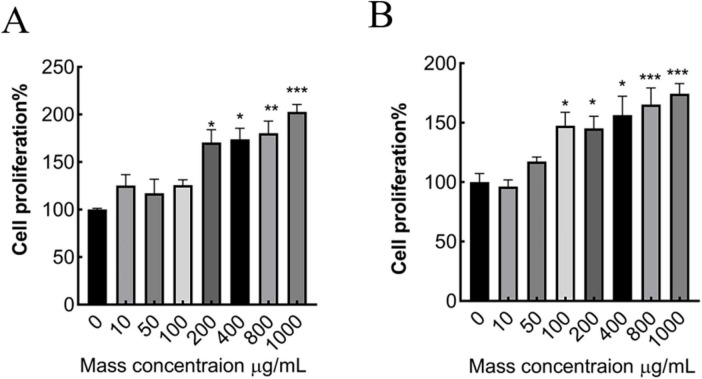
Effect of different concentrations of MMBP on MC3T3-E1 cell viability. **(A)** 24 h, **(B)** 48 h (*p < 0.05, **p < 0.01 and ***p < 0.001 compared with mass concentration of 0 μg/mL).

### 3.5 Effect of *M. miiuy* peptide on MC3T3-E1 cell proliferation

In this experiment, the relative cell growth rate was measured using a CCK8 assay. Each group was incubated with MMBP at concentrations of 10, 50, 100, 200, 400, 800, and 1,000 μg/mL for 24 and 48 h (Figure 2). Compared with the control group (without MMBP), when the concentration surpassed 100 μg/mL, preosteoblast viability increased in a dose-dependent manner. These results suggest that *M. miiuy* peptide promotes preosteoblast proliferation and has osteogenic activity.

### 3.6 Effects of MMBP on zebrafish body length and skeletal development

#### 3.6.1 Effects of MMBP on zebrafish body length

**FIGURE 3:**
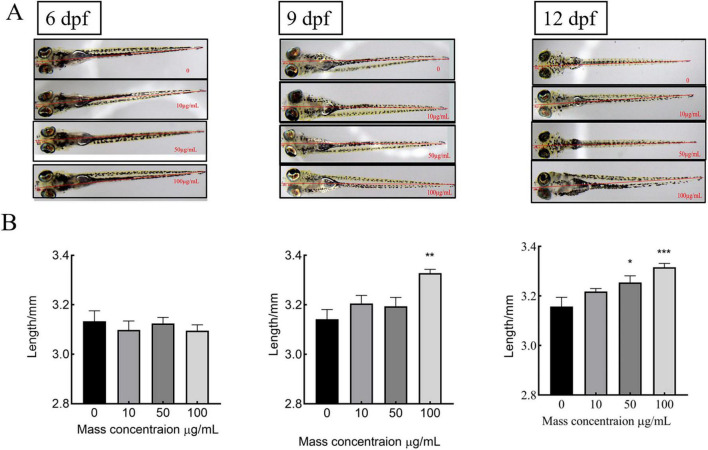
Effects of different concentrations of MMBP at 6dpf∼12dpf on the body length of zebrafish. **(A)** 6, 9, and 12 dpf using an Olympus microscope. **(B)** Graphical representation of the total body length at each dpf. All data are presented as mean ± SEM (*p < 0.05, **p < 0.01 and ***p < 0.001 compared with the mass concentration of 0 μg/mL).

To assess the effect of MMBP on growth rate, juvenile zebrafish were treated with MMBP (0, 10, 50, and 100 μg/mL) at 3 dpf and total body length was measured at 6, 9, and 12 dpf. From 6 to 9 dpf, untreated zebrafish larvae showed an increase in total body length from 3.13 ± 0.04 to 3.14 ± 0.03 mm, which persisted until 12 dpf (3.15 ± 0.04 mm). At 9 dpf, exposure to *M. miiuy* peptides at concentrations of 10, 50, and 100 μg/mL produced a notable increase in the zebrafish larvae overall body length, which measured 3.21 ± 0.03, 3.25 ± 0.03, and 3.33 ± 0.02 mm, respectively (Figure 3). Notably, the zebrafish larvae treated with the highest concentration of 100 μg/mL demonstrated a marked increase in total body length, surpassing that of untreated controls by a statistically significant margin. At 12 dpf, there was no discernible improvement in the body length of zebrafish treated with 10 μg/mL and 100 μg/mL MMBP compared to 9 dpf. Furthermore, 50 μg/mL MMBP treatment significantly increased the total body length of zebrafish at 12 dpf compared with those at 9 dpf. Therefore, MMBP may be a potential food supplement for bone formation and improved growth performance. Molagoda et al. (54) found that fermented oyster peptide extract promoted zebrafish bone formation and growth by activating the IGF-1-GSK-3β-runx2-mediated signaling pathway and upregulating growth-promoting genes, whereas the molecular mechanisms of MMBP-mediated bone formation and growth rates are not yet clear and further studies are needed to explore. these results indicate that MMBP is an effective growth stimulant.

#### 3.6.2 Effects of *M. miiuy* bone collagen peptide on skeletal development in zebrafish larvae

**FIGURE 4:**
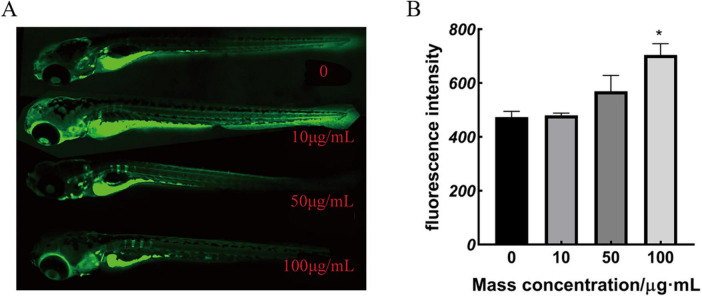
Calcium xanthophyll fluorescent dye staining results and statistical analysis of zebrafish **(A)** 9 dpf calcium xanthophyll staining, **(B)** green fluorescence intensity (*p < 0.05 compared with the mass concentration of 0 μg/mL).

There was no significant change in the green fluorescence area of 10 and 50 μg/mL-treated zebrafish compared to the control group. A notable rise in the green fluorescence area and mean optical density was observed when the applied dose was increased to 100 μg/mL. These results in Figure 4 suggest that *M. miiuy* collagen peptides promote the skeletal development of zebrafish larvae. MMBP induced an increase in the mineralized matrix in the zebrafish larvae.

### 3.7 Preventive effect of MMBP on retinoic acid–induced OP in rats

#### 3.7.1 Effect of MMBP on rat growth

**FIGURE 5:**
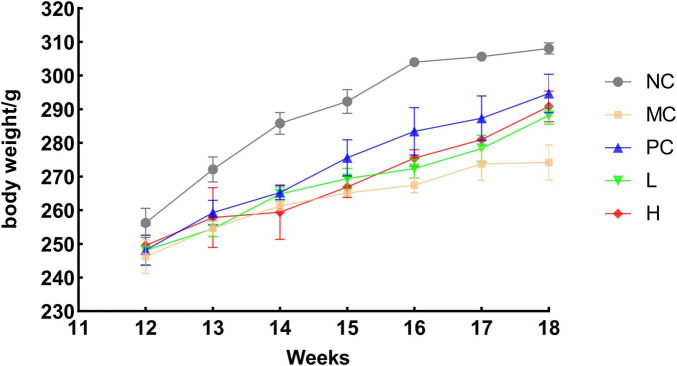
Trend of body weight growth in rats. NC, blank group, MC, model group PC, positive group L, low dose group H, high dose group.

The rats in the NC group gained weight normally. After gavage of retinoic acid while the other five groups of rats showed slower weight gain than the NC group (*p* < 0.05). The rats gained weight gradually after retinoic acid treatment was stopped, suggesting that retinoic acid reduced the rats’ appetite, resulting in decreased food intake and delayed weight gain (Figure 5). This outcome resembled that of Sun et al. (55) where after gavage of retinoic acid, the rats had a decreased appetite, decreased activity, and a significant decrease in body weight, and after treatment, the rats gained a significant amount of body weight, and the rate of increase was similar to that of the blank group.

#### 3.7.2 Effect of *M. miiuy* bone collagen peptides on rat organ

**FIGURE 6:**
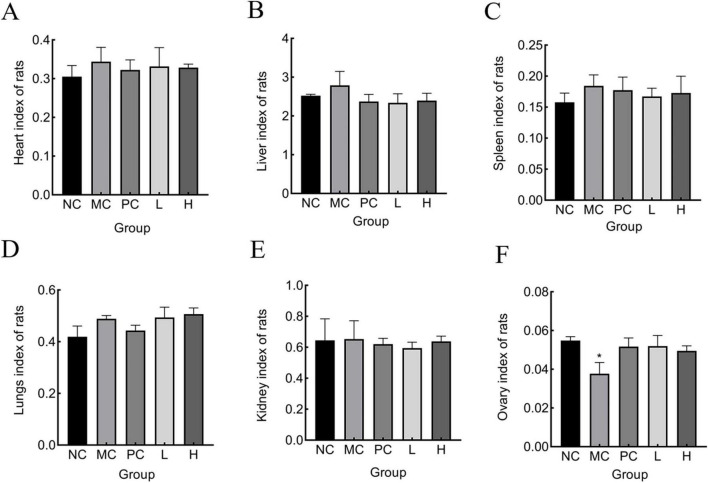
Organ index of rats. **(A)** heart, **(B)** liver, **(C)** spleen, **(D)** lung, **(E)** kidney, **(F)** ovary (*p < 0.05 compared with NC).

The organ indices of several major organs of the rats were measured to determine whether MMBP had a destructive effect, to exclude the possibility of adverse effects other than bone damage caused by retinoic acid. As shown in Figure 6, there were no notable variations in the heart, liver, spleen, and kidney of any group compared with the normal group, indicating that MMBP had no toxic effects on these five organs in rats. However, the ovarian index of the MC group was lower than that of the other groups. After MMBP intervention, the ovary weight gradually recovered, and there was no significant difference in the weights of the ovaries in the L and H groups compared with those of NC and PC groups. Retinoic acid has reproductive toxicity, destroys the gonads, disrupts the secretion of sex hormones, and causes an imbalance in bone metabolism, which in turn leads to OP. Our results suggest that ovarian injury caused by retinoic acid in rats with OP was partly alleviated by MMBP (56).

#### 3.7.3 Measurement of serum calcium, phosphonium, ALP, and TRAP

**FIGURE 7:**
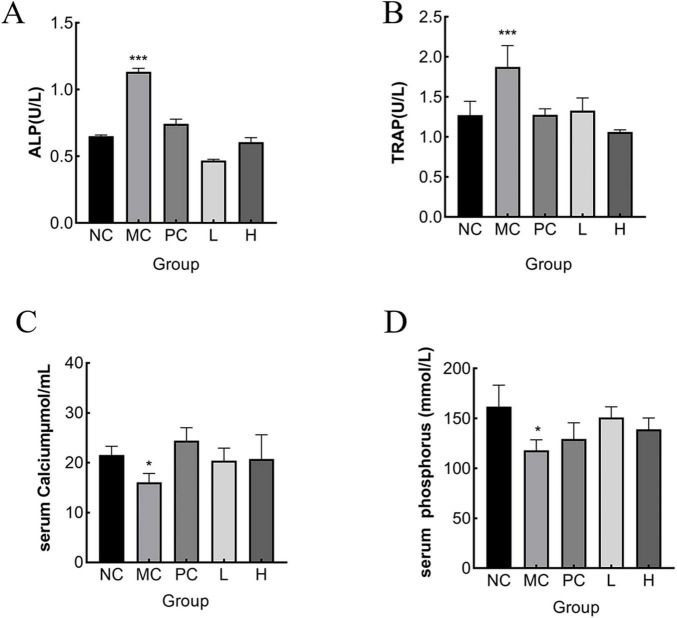
Effect of MMBP on serum indices. **(A)** ALP; **(B)** TRAP, **(C)** calcium, **(D)** phosphonium levels of rats in each group were determined (*p < 0.05, ***p < 0.01 compared with NC).

ALP is an important biochemical indicator for bone disease diagnoses. Since ALP is partly produced by osteoblasts, which promotes the combination of inorganic phosphonium and calcium, to accumulate in the bone, blood ALP activity can be a reflection of the activity of osteoblasts and the strength of bone formation (57). Osteoclasts produce TRAP, a crucial marker of osteoclast activity and bone resorption, and ver-expression leads to osteoporosis (58). The number and function of osteoclasts can be assessed by serum TRAP activity. The greater the osteoclast activity, the more bone was lost (59). However, the ability of bone formation to effectively counteracts for this loss, bone tissue was destroyed, making bone density lower and bones weaker and more fragile (60). As shown in Figures 7A, B, serum ALP and TRAP activity levels were notably elevated (*p* < 0.05) in the NC group compared to that of the MC group. This may be due to the stimulatory effect of retinoic acid on the increased activity of osteoblasts and osteoclasts, leading to the development of high-conversion OP. Elevated serum ALP levels may also be associated with abnormal calcium absorption. Compared with the MC group, MMBP treatment significantly reduced serum ALP and TRAP activities (*p* < 0.05), indicating that MMBP could effectively reduce the bone conversion rate and improve calcium absorption to normal levels. Rat serum calcium and phosphonium levels are displayed in Figures 7C, D. It is evident that the bone serum calcium and phosphonium levels in the MC group were much lower than those in the NC group (*p* < 0.05). The findings indicated an imbalance in calcium and P metabolism, resulting in considerable losses of calcium and phosphonium in the MC group. Additionally, the group receiving MMBP treatment had considerably higher serum calcium and phosphonium levels (*p* < 0.05) than the NC group. These findings indicate that MMBP therapy successfully reduces the bone mineral loss triggered by retinoic acid and enhances bone quality.

#### 3.7.4 Effect of MMBP on BMD in rats

**FIGURE 8:**
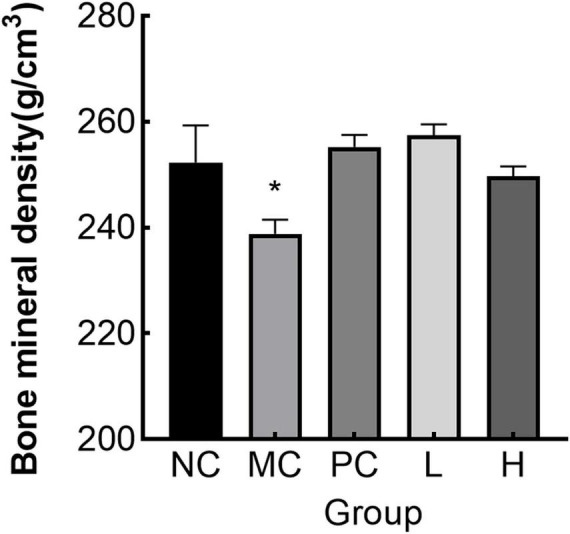
Bone mineral density (BMD) in rats (*p < 0.05 compared with NC).

The MC group had considerably lower femoral BMD (*p* < 0.05) than the NC group, as illustrated in Figure 8. The L and H groups had considerably higher femoral BMD (*p* < 0.05) than the MC group. The decline in BMD in osteoporotic rats caused by retinoic acid was verified in earlier research (31). As demonstrated by our BMD analysis results, MMBP had a beneficial effect on bone formation and could partially repair the BMD of osteoporotic rats.

Retinoic acid not only activated osteoclast activity and promoted bone resorption, but also activated osteoblast activity, resulting in a high bone conversion rate in the organism, but overall bone resorption was greater than bone formation (61). ALP was a marker of bone formation and TRAP was a marker of bone resorption (62). In this experiment, ALP and TRAP activities in the model group were significantly higher than those in the administered group and the normal group, and both L and H were able to significantly reduce ALP and TRAP activities, indicating that MMBP could effectively inhibit the phenomenon of high bone conversion caused by retinoic acid. Retinoic acid can reduce the bone density, decrease the bone inorganic mass, make the bone trabeculae thinner and fewer in number, and increase the risk of fracture (63). Compared with the normal group, the BMD content of the model group was significantly reduced, indicating the success of modeling. And the BMD of each dosing group was higher than that of the MC group after the administration of retinoic acid, and the results indicated that MMBP was able to increase the bone mass and improve the BMD. Recently, studies have shown progress in managing OP through natural products. Xia et al. (13) et al. found that phosphorylated peptides from Antarctic krill Euphausia superba could ameliorate mouse denuded osteoporosis by increasing the OPG/RANKL ratio and thus inhibiting the activation of the NF-κB signaling pathway. IERGDVVVVQDSPSD and RGDLGIEIPTEK extracted from the yellow croaker have been shown to have significant effects in promoting osteoblast proliferation and increasing ALP activity (64). Overall, naturalized active peptides are becoming increasingly important in the design of anti-osteoporosis products. The ability is due to collagen peptides can supplement bone organic components, so that the collagen fiber mesh structure in the bone is tight and complete, not only for calcium and phosphorus mineralization to form bone inorganic material to provide a good place, but also can effectively fix the organic matter, thus preventing the loss of organic matter (65). At the same time, since collagen peptides also had the ability to chelate calcium, their dual action made them play an important role in helping to strengthen bones and promote calcium absorption (7). The potential efficacy of collagen peptides in preventing and improving osteoporosis has been demonstrated, which provides a solid theoretical basis for the development of food-borne peptide calcium supplementation products using *M. miiuy* and also provides new ideas and directions for research and development in related fields.

## 4 Conclusion

This study systematically analyzed the nutritional composition of *M. miiuy* bones and showed that *M. miiuy* bones are a rich source of protein, calcium, selenium, and P, whereas their fat content is low. This study showed that MMBP had a favorable effect in promoting osteoblast proliferation. MMBP had high affinity (PDB: 2H62) with BMPAR1, which promoted MC3T3-E1 cell proliferation, zebrafish skeletal development and growth, and BMD improvement in rats. MMBP can maintain serum ALP level and increases BMD, which can promote bone formation and prevent resorption. Fish bone can be further developed into a leisure food, a calcium supplement, or a flavor base. Using this byproduct not only benefits the environment by reducing waste, but it would also have economic benefits, thereby providing theoretical support for additional studies on the comprehensive utilization of *M. miiuy* bone.

## Data Availability

The original contributions presented in the study are included in this article/Supplementary material, further inquiries can be directed to the corresponding authors.

## Footnotes

1
http://distilldeep.ucd.ie/peptideranker/

2
http://distilldeep.ucd.ie/cpppred/

3
http://crdd.osdd.net/raghava/toxinpred/

4
https://www.ddg-pharmfac.net/allertop/index.html

5
http://www.innovagen.com

6
https://biochemia.uwm.edu.pl/biopep-uwm/

7
http://web.expasy.org/compute_pi/

8
http://www.tulane.edu/∼biochem/ww/pepdraw

9
http://web.expasy.org/protparam/

10
http://www.rcsb.org/pdb
