# Supplementary material for: Evaluation of nutrient composition and bone-promoting activity of miiuy croaker (Miichthys miiuy) bone
Source: Front Nutr. 2024 Dec 31;11:1510028. doi: 10.3389/fnut.2024.1510028 (PMC11729392; doi:10.3389/fnut.2024.1510028)
Supplement: Supplementary file 1 [file Data_Sheet_1.docx]

**This docx file includes:**

**Supplementary Methods**

**Tables S1 to S4**

**Table S1.** Amino acid mass fraction of Miichthys Miiuy bone (g/100 g).

**Table S2.** Amino acid score and chemical score of Miichthys Miiuy bone.

**Table S3.** Predicted biological activities of peptides.

**Table S4.** Molecular docking results.

**FigureS1.** LC-MS analysis of total ion flow chromatogram of collagen peptides from Miichthys Miiuy bone peptide.

Table S1 Amino acid mass fraction of *Miichthys Miiuy* bone (g/100 g)

| Amino acids | Quantity contained |
| --- | --- |
| Phenylalanine (Phe) | 0.51 |
| Alanine (Ala) | 2.53 |
| Methionine (Met) | 0.48 |
| Proline (Pro) | 3.11 |
| Glycine (Gly) | 5.48 |
| Glutamic acid (Glu) | 2.42 |
| Lysine (Lys) | 0.88 |
| Serine (Ser) | 0.67 |
| Threonine (Thr) | 0.68 |
| Aspartic acid (Asp) | 1.40 |
| Valine (Val) | 0.63 |
| Arginine (Arg) | 1.97 |
| Tyrosine (Tyr) | 0.22 |
| Leucine (Leu) | 0.65 |
| Isoleucine (Ile) | 0.27 |
| Histidine (His) | 0.23 |
| ∑EAA | 4.33 |
| ∑NEAA | 17.8 |
| ∑EAA/∑TAA | 19.57 |
| ∑EAA/∑NEAA | 24.32 |
| ∑FAA | 11.83 |
| TAA | 22.13 |

Note: ∑EAA: total essential amino acids; ∑NEAA total non-essential amino acids; TAA: total amino acids; ∑FAA total fresh flavor amino acids

Table S2 Amino acid score and chemical score of Miichthys Miiuy bone

| Amino acids | Egg | AAS | CS |
| --- | --- | --- | --- |
| Isoleucine (Ile) | 331 | 0.25 | 0.19 |
| Leucine (Leu) | 534 | 0.34 | 0.28 |
| Lysine (Lys) | 441 | 0.60 | 0.46 |
| Threonine (Thr) | 292 | 0.63 | 0.54 |
| Valine (Val) | 411 | 0.47 | 0.35 |
| Lorine + Phenylalanine (Thr+Phe) | 565 | 0.44 | 0.30 |

Table S3 Predicted biological activities of peptides

| Peptide Sequence | Peptide Ranker | CPPpred | Theoretical pI |
| --- | --- | --- | --- |
| GAPGPAGPRGPA | 0.833943 | 0.271967 | 9.75 |
| GPAGAPGPAGPRGPA | 0.888421 | 0.285693 | 9.75 |
| GPAGPRGPAGPHGPA | 0.857516 | 0.235941 | 9.76 |
| GPAGPTGSVGRP | 0.685213 | 0.2151 | 9.75 |
| GRVGPPGPSGVA | 0.500379 | 0.214172 | 9.75 |
| GPAGPRGPAGPH | 0.792461 | 0.211629 | 9.76 |
| GPHGPAGPRGPAGPHGPAGKD | 0.801446 | 0.243553 | 8.76 |
| GARGAPGPAGPR | 0.712702 | 0.417848 | 12 |
| GPRGPPGPA | 0.80939 | 0.25499 | 9.75 |
| GKNGDRGETGPAGPSGAPGPAGPRGPA | 0.648411 | 0.305879 | 8.75 |
| GPAGPRGPAGPHGPAGKD | 0.807855 | 0.250887 | 8.75 |
| GPAGARGADGNVGPA | 0.564368 | 0.22779 | 5.84 |
| GAAGPAGPRGPAGS | 0.713455 | 0.221011 | 9.75 |
| AGPAGPRGPAGSA | 0.74732 | 0.226807 | 9.79 |
| GARGDRGFPGER | 0.578412 | 0.27237 | 9.51 |
| GPAGPRGPAGAV | 0.770912 | 0.299454 | 9.75 |
| GPAGKNGDRGETGPAGPSGAPGPAGPR | 0.643226 | 0.289576 | 8.75 |
| GGRGNEGPQGARGEPGNPGPSGPAGPA | 0.664303 | 0.226388 | 6.14 |
| GSPGPAGPRGPQGL | 0.899585 | 0.207061 | 9.75 |
| GTDGAPGKDGPRGL | 0.600832 | 0.226863 | 5.96 |
| GPQGPAGPPGPKGARGGA | 0.936193 | 0.329641 | 11 |
| GAAGPAGPRGPA | 0.771751 | 0.298035 | 9.75 |
| GAAGQRGL | 0.536739 | 0.399925 | 9.75 |
| GPSGPRGAPGERGETGPAGPA | 0.654922 | 0.227908 | 6.14 |
| GRAGPAGPAGAR | 0.709242 | 0.437764 | 12 |
| GPAGPVGKDGARGA | 0.69622 | 0.283187 | 8.75 |

Table S4 Molecular docking results

| Peptide Sequence | Affinitykcal/mol | Molecular Weight | Instability index | Net charge |
| --- | --- | --- | --- | --- |
| GAPGPAGPRGPA | -8.9 | 1003.5199 | 36.88 | 1 |
| GPAGAPGPAGPRGPA | -8.6 | 1228.6312 | 44.35 | 1 |
| GPAGPRGPAGPHGPA | -8.3 | 1294.653 | 30.25 | 1 |
| GPAGPTGSVGRP | -8.3 | 1051.541 | 27.12 | 1 |
| GRVGPPGPSGVA | -8.2 | 1049.5618 | 34.19 | 1 |
| GPAGPRGPAGPH | -8.1 | 1069.5416 | 27.91 | 1 |
| GPHGPAGPRGPAGPHGPAGKD | -8 | 1885.9294 | 15.49 | 1 |
| GARGAPGPAGPR | -8 | 1062.5682 | 13.76 | 2 |
| GPRGPPGPA | -7.9 | 1105.5338 | 33.88 | 1 |
| GKNGDRGETGPAGPSGAPGPAGPRGPA | -7.9 | 2384.158 | 15.63 | 1 |
| GPAGPRGPAGPHGPAGKD | -7.8 | 1594.7964 | 22.16 | 1 |
| GPAGARGADGNVGPA | -7.8 | 1265.6112 | 1.05 | 0 |
| GAAGPAGPRGPAGS | -7.8 | 1121.5577 | 19.29 | 1 |
| AGPAGPRGPAGSA | -7.7 | 1064.5363 | 26.53 | 1 |
| GARGDRGFPGER | -7.7 | 1273.6276 | -8.57 | 1 |
| GPAGPRGPAGAV | -7.4 | 1005.5355 | 20.83 | 1 |
| GPAGKNGDRGETGPAGPSGAPGPAGPR | -7.3 | 2384.158 | 18.77 | 1 |
| GGRGNEGPQGARGEPGNPGPSGPAGPA | -7.2 | 2397.1169 | 30.29 | 0 |
| GSPGPAGPRGPQGL | -7.1 | 1246.6417 | 56.74 | 1 |
| GTDGAPGKDGPRGL | -7.1 | 1296.6422 | -6.6 | 0 |
| GPQGPAGPPGPKGARGGA | -6.9 | 1527.7905 | 29.53 | 2 |
| GAAGPAGPRGPA | -6.9 | 977.5042 | 20.83 | 1 |
| GAAGQRGL | -6.5 | 728.3929 | 12.48 | 1 |
| GPSGPRGAPGERGETGPAGPA | -6.4 | 1873.903 | 19.27 | 0 |
| GRAGPAGPAGAR | -6.4 | 1036.5526 | 34.19 | 2 |
| GPAGPVGKDGARGA | -6.3 | 1208.6261 | 6.48 | 1 |


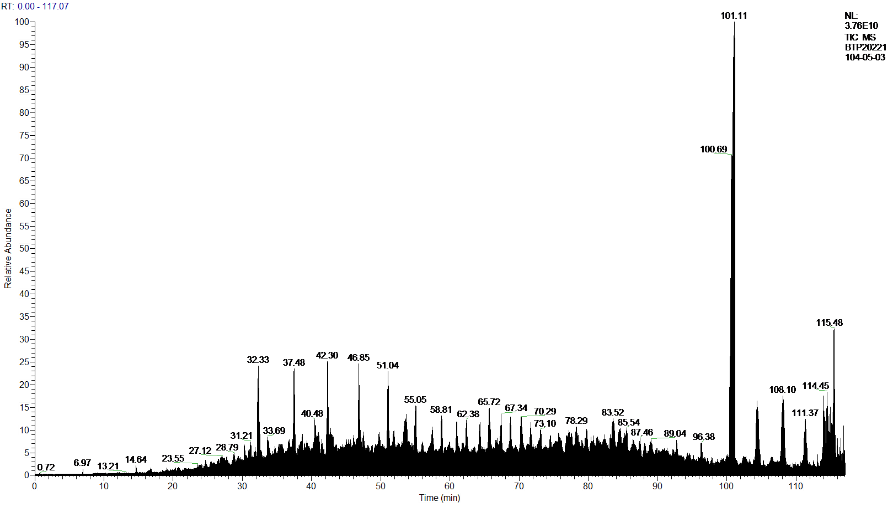


Figure S1. LC-MS analysis of total ion flow chromatogram of collagen peptides from Miichthys Miiuy bone peptide.
